# A tri-layer decellularized, dehydrated human amniotic membrane scaffold supports the cellular functions of human tenocytes in vitro

**DOI:** 10.1007/s10856-023-06740-4

**Published:** 2023-07-24

**Authors:** Yong Mao, Nikita John, Nicole M. Protzman, Desiree Long, Raja Sivalenka, Shamshad Azimi, Brandon Mirabile, Robert Pouliot, Anna Gosiewska, Robert J. Hariri, Stephen A. Brigido

**Affiliations:** 1 Laboratory for Biomaterials Research, Department of Chemistry and Chemical Biology, Rutgers University, 145 Bevier Rd., Piscataway, NJ 08854 USA; 2 Healthcare Analytics, LLC, 78 Morningside Dr, Easton, PA 18045 USA; 3 Celularity Inc., 170 Park Ave., Florham Park, NJ 07932 USA

## Abstract

Differences in scaffold design have the potential to influence cell-scaffold interactions. This study sought to determine whether a tri-layer design influences the cellular function of human tenocytes in vitro. The single-layer decellularized, dehydrated human amniotic membrane (DDHAM) and the tri-layer DDHAM (DDHAM-3L) similarly supported tenocyte function as evidenced by improved cell growth and migration, reduced dedifferentiation, and an attenuated inflammatory response. The tri-layer design provides a mechanically more robust scaffold without altering biological activity.

**Graphical Abstract:**

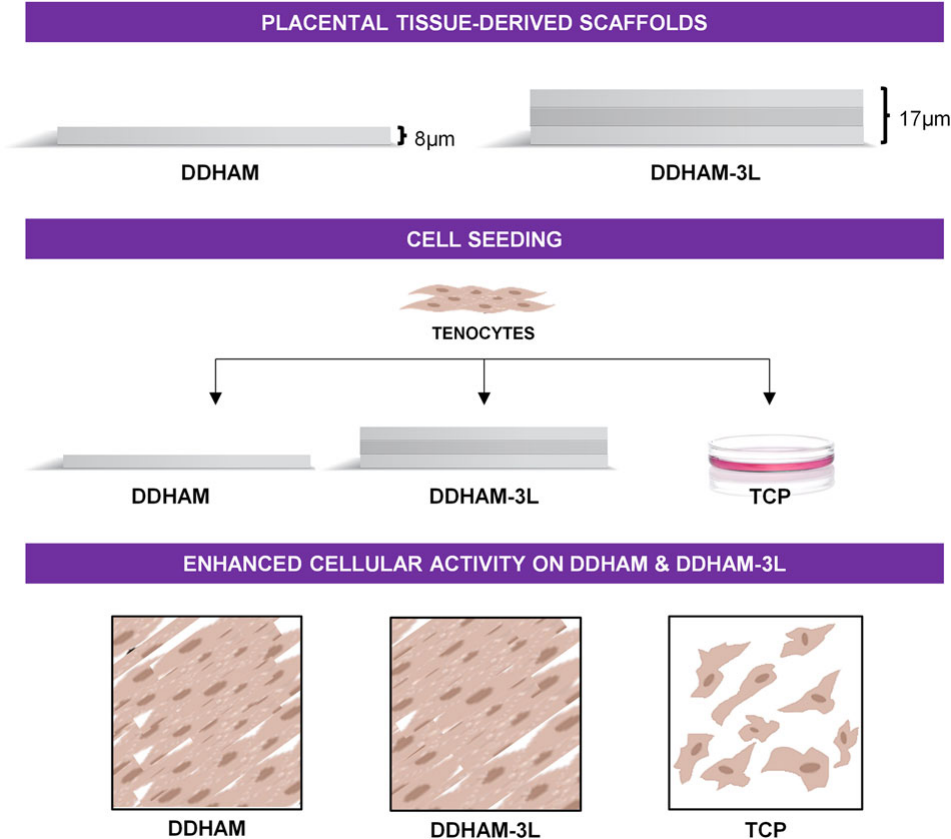

## Introduction

Tendons are unique forms of dense connective tissue that connect muscle to bone and transmit forces to produce movement. Tendons are prone to injury, which often occurs secondary to overuse, traumatic injury, or intrinsic age-related degeneration. Due to the combined effects of population growth, increased life expectancy, and heightened participation in extreme/competitive sports, tendon injuries have become increasingly common [1–5].

The hypocellularity and hypovascularity of tendons contributes to a slow and ineffective healing process [6]. The repaired tendon is burdened by misaligned collagen fibers, a disorganized extracellular matrix (ECM), and scar tissue formation [7]. As a result, the structural and biomechanical properties of the healed tendon are inferior to that of the native tendon. This loss of mechanical competence is associated with a high risk of tendon degeneration and re-rupture [8, 9]. The need for more advanced treatment strategies, capable of addressing the underlying pathology, is evident.

Tenocytes are the resident cells found in tendon tissue and account for 90% to 95% of the cellular composition [10]. These cells synthesize ECM components, including collagen (primarily types I [11], III [11], V [11], and VI [12]), proteoglycans, and glycoproteins [6, 10] and have been characterized by the expression of tenogenic differentiation genes, including scleraxis (*SCX*) [13, 14], tenomodulin (*TNMD*) [15, 16], tenascin-C (*TNC*) [17], and types I and III collagen (*COL1A1* and *COL3A1*, respectively [18, 19]. Tenocytes are also responsible for tissue maintenance, tissue remodeling, and tissue repair. As such, they respond to and modulate pro-inflammatory cytokines, such as interleukin-1 beta (IL-1β), interleukin-8 (IL-8), and tumor necrosis factor alpha (TNF-α) [20]. The cells have also been shown to regulate the production of inflammation modulator and pro-fibrotic factor, transforming growth factor beta-1 (TGF-β1), anti-fibrotic factor, transforming growth factor beta-3 (TGF-β3) [21–24], and matrix metalloproteinases, such as matrix metallopeptidase-1 (MMP-1), a regulator of matrix turnover [25]. For this reason, it is important to examine how new tissue engineering and regenerative medicine treatment strategies modulate tenocyte behavior.

Scaffolds represent a promising means to modulate tenocyte behavior and in turn tendon healing [26–29]. Due to their mechanical properties and biological activities, scaffolds augment tendon repair by providing both structural and cellular support [30]. To promote rapid integration without inducing an immune response, an ideal scaffold must exhibit excellent biocompatibility and biodegradability [28, 31]. Once implanted the material should mimic the ECM of the surrounding environment, permit cell-biomaterial interactions, and promote cell adhesion, proliferation, migration, and differentiation [28, 31, 32]. Previous research has shown that an ECM can reduce the dedifferentiation of primary cells [33–35] and modulate inflammatory gene expression to support tendon healing [25, 35].

Several types of scaffolds are commercially available for the augmentation of tendon repair and have demonstrated varying degrees of success [26, 28, 29]. Given the structural, biochemical, and immunogenic properties of placental tissue [36–38], scaffolds derived from the placenta are a unique subset of biological scaffolds with a promising application in tendon repair [35, 39]. A recent in vitro report confirmed that placental tissue-derived ECMs support tenocyte proliferation with reduced de-differentiation and attenuation of the inflammatory response [35]. However, even within this unique subset of scaffolds, products vary in terms of tissue source, processing methodologies, decellularization, and product design.

The purpose of this study is to determine whether a tri-layer design influences the cellular function of human tenocytes in vitro. Therefore, two placental tissue-derived scaffolds were selected: (1) a decellularized, dehydrated human amniotic membrane (DDHAM); and (2) a tri-layer decellularized, dehydrated human amniotic membrane (DDHAM-3L). The two placental tissue-derived scaffolds are sourced from the same tissue and undergo the same processing and decellularization protocols with one marked difference in product design, the number of layers. The increased layering is designed to enhance the thickness and handling properties of the scaffold [40].

Both placental tissue-derived amniotic scaffolds are excised from qualified term placentas, washed, and scraped to remove extraneous tissues and cells. The scaffolds are then decellularized using an osmotic shock, followed by a mild detergent treatment. They are then dried and sterilized. Previous research has confirmed that this proprietary decellularization process removes residual cells, cell debris, growth factors, and cytokines, while retaining an ECM structure with high collagen content and key bioactive molecules, such as fibronectin, laminin, glycosaminoglycans, and elastin [38]. DDHAM-3L has a three-layer configuration to improve handleability for surgical application [40]. It is also uniquely designed with an all-sided stromal interface [40].

A recent in vitro comparison demonstrated that differently designed ocular amniotic membranes influence the activity and inflammatory response of human corneal epithelial cells [40]. Given these previous results [40], we hypothesize that differences in product design may alter tenocyte growth, migration, dedifferentiation, and inflammatory response.

This study aims to evaluate the following cellular responses of tenocytes on DDHAM and DDHAM-3L:

I. Growth,
II. Migration,
III. Dedifferentiation, and
IV. Inflammatory response under resting or TNF-α-stimulated conditions.

## Materials and methods

It has been determined that this research does not require Institutional Review Board approval, since there is no intervention or interaction with human subjects.

### Scaffolds

Two placental tissue-derived scaffolds were selected: DDHAM and DDHAM-3L (Celularity Inc., Florham Park, NJ). Both placental tissue-derived scaffolds are processed from human tissue according to the American Association of Tissue Banks (AATB) standards, and are regulated as a human cell, tissue, or cellular or tissue-based product (HCT/P) under Section 361 of the Public Health Service Act and 21 CFR Part 1271. Amniotic tissue is harvested from the placental disk and skirt which is then subjected to multiple rounds of cleaning, rinsing, and cell scraping. The amniotic membrane undergoes mechanical scraping intended to remove nearly 100% of the amniocytes and chorionic cells from the surface of the membrane. Most of the fibroblasts are also removed from the substance of the tissue. Next, the decellularized tissue is dehydrated and then cut into the desired size to form a single-layer DDHAM. Alternately, the DDHAM-3L is layered onto itself and dried to create a tri-layer amniotic membrane sheet.

### Scaffold characterization

Scaffold characterization measurements included scaffold thickness, width, maximum force, and tensile strength. Biomaterial samples were cut into standardized 2 cm by 3 cm rectangular strips, using a #11 scalpel (Integra, Plainsboro, NJ). Care was taken to ensure uniformity in sample size and shape. Testing was performed using a Mark-10 F105-IMT motorized test stand, equipped with a Mark-10 FS05-50 digital force gauge (Copiague, NY). Uniaxial tension was applied until failure (i.e., the biomaterial sample ruptured). The digital force gauge was calibrated before each test to ensure accurate measurements. One end of each biomaterial sample was securely attached to the pneumatic grip of the Mark-10 stand, gripping approximately 1 cm of the specimen length. The other end was connected to a second pneumatic grip, connected to the digital force gauge, ensuring a secure and reliable connection. Dry biomaterial samples were pulled in tension at a rate of 1 mm/min until the sample ruptured. The maximum force at the point of failure was recorded as the tensile strength of the biomaterial. The test was repeated twice with two samples to ensure reliability. Scaffold thickness and width in millimeters (mm) were recorded prior to testing and were used in the conversion of the raw force data, measured in Newtons (N), into units of tensile strength measured in Megapascals (MPa). The recorded data, including maximum force values, were analyzed to calculate average tensile strength.

Histological staining, using a standard Hematoxylin and Eosin (H&E) staining assay, was also performed on DDHAM and DDHAM-3L to evaluate the effect of layering on tissue morphology and to confirm the absence of any residual cells and cell debris. DDHAM and DDHAM-3L lots were cut into 1 cm by 2 cm pieces for each lot and sent to siParadigm (Pine Brook, NJ) for slide preparation and staining. Samples were dehydrated, infiltrated with paraffin, and embedded into paraffin blocks, which were cut into thin sections and mounted on slides. Slides were de-waxed with xylene and washed with alcohol and water. Samples were stained with Hematoxylin nuclear stain and Eosin stain, and rinsed with alcohol and water, as well as xylene. H&E-stained slides from siParadigm were evaluated by microscopic examination and imaging using Imaging system Axio Observer.A1 (Zeiss, Oberkochen, Baden-Württemberg, Germany).

### Primary cells

Human tenocytes were purchased from ZenBio Inc. (Research Triangle, NC). Cells were cultured in tenocyte medium (ZenBio Inc., Research Triangle, NC), following the manufacturer’s instructions.

### Preparation of scaffolds

10 mm discs of scaffolds were punched directly from DDHAM and DDHAM-3L, using a 10 mm biopsy punch (Acuderm Inc 10 mm Biopsy punch Cat# NC9236770, Thermo Fisher Scientific, Waltham, MA). Samples from 2–3 different batches (donors) were included for each individual experiment.

### Assessment of cell growth on different scaffolds

10 mm discs of each scaffold sample were placed in the wells of four 48-well plates (Cell-Repellent 48-Well Microplate, Greiner Bio-One, Monroe, NC). A sterile O-ring (Chemical-Resistant Viton® Fluoroelastomer O-ring, McMaster-Carr, Robbinsville, NJ), measuring 2 mm in width and 7 mm in diameter, was placed on top of the scaffold to hold the scaffold in place. A sterile O-ring was also placed in the wells of the 48-well TCP plate. 0.4 mL/well of complete growth medium (ATCC, Manassas, VA) was added to each well. To condition the scaffolds, the 48-well plates were incubated at 37 °C for 2 h (h).

Human tenocyte cells at passage 3–4 (P3-4) were cultured to 80% confluence in 10 cm cell culture dishes, following the manufacturer’s instructions. Cells were rinsed once with 5 mL of phosphate-buffered saline (PBS) per dish. 1 mL of 0.25% trypsin (Gibco, Thermo Fisher Scientific, Waltham, MA) was added to each dish and incubated at 37 °C for 5 min (min). 2 mL of culture medium (DMEM + 10% FBS + Penicillin/Streptomycin, Thermo Fisher Scientific, Waltham, MA) were added to the dish to neutralize the trypsin. Cells were transferred to 15 mL conical tubes and centrifuged at 240 × *g* for 5 min. Cells were re-suspended in complete growth medium and counted using a hemocytometer.

Tenocytes (2 × 10^4^/well) were added to each well containing a scaffold. As a control, cells were added to wells of 48-well tissue culture treated plastic (TCP). The plates were incubated at 37 °C with 5% CO_2_ and 95% humidity at an atmospheric O_2_ concentration of 20% for 1, 2, 4, and 7 days. After incubation for 24 h (Day 1, the first time point), the medium from each well of all plates was removed and fresh medium was added. The number of cells in the first set of plates was measured using alamarBlue assay (Bio-Rad, Hercules, CA). Briefly, 0.2 mL/well of alamarBlue solution (complete growth medium+10% alamarBlue reagent) was added to each well and incubated at 37 °C for 30 min. After incubation, 0.1 mL/well of supernatant was transferred to a 96-well plate. Fluorescent intensity was read using a multimode microplate reader (Spark®, TECAN, Switzerland) at excitation/emission (Ex/Em) = 540 nm/590 nm. The fluorescent intensity was expressed in arbitrary units (AU). To convert the fluorescent intensity to number of cells, a standard curve was determined, as follows. Tenocytes were seeded onto TCP at different densities (serially diluted (1:2) from 40,000 cells/well to 313 cells/well) and cultured for 24 h. A linear curve was obtained by graphing fluorescent intensity (AU) and cell number (R^2^ > 0.99, data not shown). The equation of this curve was used to convert fluorescent intensity (AU) to cell number for testing samples. The second, third, and fourth sets of plates were cultured at 37 °C for 2, 4, and 7 days. At each time point, the number of cells in the designated plate was determined using alamarBlue assay, as described above.

### Live staining of cells

Tenocytes were cultured on the scaffolds, and the medium was changed every three days. On Day 7, the medium was removed from each well, and 0.2 mL/well of fresh complete growth medium containing 50 nM Calcein AM (Thermo Fisher Scientific, Waltham, MA) was added to each well. After incubation for 30 min at 37 °C, the medium was removed. Cells were washed twice with PBS and observed under epi-fluorescent microscope (Zeiss Axio Observer D1, Jena, Germany).

### Conditioned media for migration assay

Tenocytes (2 × 10^4^/well) were seeded onto scaffolds. After culturing for 24 h, the medium was removed. 0.45 mL/well of migration medium (complete culture medium diluted 1:1 with Dulbecco’s Modified Eagle Medium (DMEM, Thermo Fisher Scientific, Waltham, MA) base medium to reduce FBS concentration to 5%) was added to each well and incubated at 37 °C for 24 h. The supernatants (24 h CM) were collected from each well and stored at −80 °C until being used in the migration assay.

### Transwell migration assay

0.45 mL of conditioned medium (CM) was transferred (collected as described above) to each well of a 24-well plate. A transwell insert (6.5 mm diameter, 8.0 µm pore size PET membrane, Costar, Corning, NY) was added to each well containing CM. Tenocytes were resuspended in DMEM base medium (no FBS). 0.3 mL of tenocytes (1 × 10^5^) were added to each transwell insert and incubated at 37 °C with 5% CO_2_ and 95% humidity. After incubation for 20 h, the cells were carefully removed from the inside of each insert using kim-wipe paper (Thermo Fisher Scientific, Waltham, MA) and the inserts were transferred into a new 24-well plate containing 0.4 mL/well MTT solution (MTT (3-(4,5-Dimethylthiazol-2-yl)-2,5-Diphenyltetrazolium Bromide, M6494, Thermo Fisher Scientific, Waltham, MA)) (1 mg/mL in DMEM base medium) and incubated for 1 h. The inserts were rinsed in PBS and each insert was immersed in 0.3 mL/well of extraction solution (0.04 N Hydrochloric acid (HCl) in isopropanol (Sigma-Aldrich, Burlington, MA) in a fresh 24-well plate. The plate was rocked to mix and extract the MTT. 100 µL of extractants were transferred to a well of 96-well plate, and the absorbance was read at 570 nm (650 nm served as reference), using a multimode microplate reader (Spark®, TECAN, Switzerland). Migration is expressed as the percentage of migrated cells to the total number of cells (OD_sample_/OD_control_ × 100%).

### Preparation of ribonucleic acid (RNA) lysates for assessment of tenocyte dedifferentiation

Tenocytes (2 × 10^4^/well) were added to each well of 48-well plates containing scaffolds. There were two sets of samples. After culturing for 24 h, the medium was removed from all samples. Fresh culture medium was added to each well and incubation continued. After 24 h, the medium was removed from each well of one set of samples. Cells in the wells were lysed with 0.2 mL/well RNA Lysis buffer (Promega, Durham, NC). The RNA lysates were stored at −80 °C. The cells in the second set of samples continued to culture, and the medium was changed every three days. On Day 7, the medium was removed from each well of the second set of samples. Cells in the wells were lysed with 0.2 mL/well RNA Lysis buffer.

### Stimulation of tenocytes with TNF-α

Tenocytes (2 × 10^4^/well) were added to 48-well plates containing scaffolds. After culturing for 24 h, the medium was removed. 0.5 mL/well of fresh growth medium (−) or 0.5 mL/well of fresh medium + 10 ng/mL human TNF-α (+) (PeproTech, Rocky Hill, NJ) was added and incubated at 37 °C. After 24 h, cells were lysed with 0.2 mL/well RNA Lysis buffer. The RNA lysates were either stored at −80 °C to be used later or used immediately for RNA isolation.

### Primers for quantitative polymerase chain reaction

QuantiTect primers used in quantitative polymerase chain reaction (qPCR) were purchased from Qiagen (Germantown, MD; Table 1).

**Table 1:** Primers for quantitative polymerase chain reaction. The genes and primers used for qPCR are listed

Gene	Gene Symbol	Primer
Glyceraldehyde-3-Phosphate Dehydrogenase	*GAPDH*	Hs_GAPDH_2_SG QT01192646
Scleraxis	*SCX*	Hs_SCX_2_SG QT01529507
Tenascin C	*TNC*	Hs_TNC_1_SG QT00024409
Collagen Type I Alpha 1	*COL1A1*	Hs_COL1A1_1_SG QT00037793
Collagen, Type III, Alpha 1	*COL3A1*	Hs_COL3A1_1_SG QT00058233
Tumor Necrosis Factor	*TNF*	Hs_TNF_3_SG QT01079561
C-X-C Motif Chemokine Ligand 8	*CXCL8*	Hs_CXCL8_1_SG QT00000322
Transforming Growth Factor Beta 1	*TFGβ1*	Hs_TGFB1_1_SG QT00000728
Transforming Growth Factor Beta 3	*TFGβ3*	Hs_TGFB3_1_SG QT00001302
Matrix Metallopeptidase 1	*MMP1*	Hs_MMP1_1_SG QT00014581

### Quantification of the relative expression of mRNA by qPCR

The quantification of the relative expression of tenocyte phenotypic genes and inflammatory markers was performed in tenocytes cultured on scaffolds by qPCR, as previously described [4]. Briefly, total RNA from these lysates was purified using SV 96 Total RNA Isolation System (Promega, Madison, WI). RNA concentration and purity were measured using TECAN Spark Nano plate (TECAN, Switzerland). cDNA preparation and qPCR were performed on Roche Lightcycler 480 following a standard procedure. qPCR was carried out for 45 cycles. Each amplification cycle was set as follows: 95 °C for 10 s with 4.4 °C/s ramp, 60 °C for 10 s with 2.2 °C/s ramp, 72 °C for 10 s with 4.4 °C/s ramp. Every testing condition has 3–4 biological repeat samples, and each sample was run in duplicate. After the run was completed, a second derivative analysis was performed using the raw data to determine the mean Cp (Crossing point-PCR-cycle) for each sample. GAPDH was chosen to be the reference gene based on previous reports [41, 42]. mRNA expression relative to *GAPDH* was determined by Pfaffl analysis (2ΔCp target/2ΔCp reference), in which ΔCp = mean Cp of sample – mean Cp of the cells of Day 0 (starting cells as reference) [33].

### Statistical analysis

Each independent experiment contained 3 or more biological repeat samples (*n* ≥ 3) as indicated by each individual experiment. Data are presented as the mean ± standard deviation. Results are representative of at least two independent experiments, except for the transwell migration assay which was performed once. Analyses were conducted using IBM SPSS (Build 1.0.0.1444). The data were tested and found to be approximately normally distributed. One and two-way analysis of variance (ANOVA) with Tukey post-hoc tests were conducted. Significant interactions were evaluated with simple main effects analysis with Sidak correction for multiple comparisons. Independent sample *t*-tests were conducted when there were two samples. The significance level for all statistical tests was set at *p* = 0.05. Artwork was created using Microsoft Excel (Version 2210).

## Results

### Scaffold characterization

**Fig. 1:**
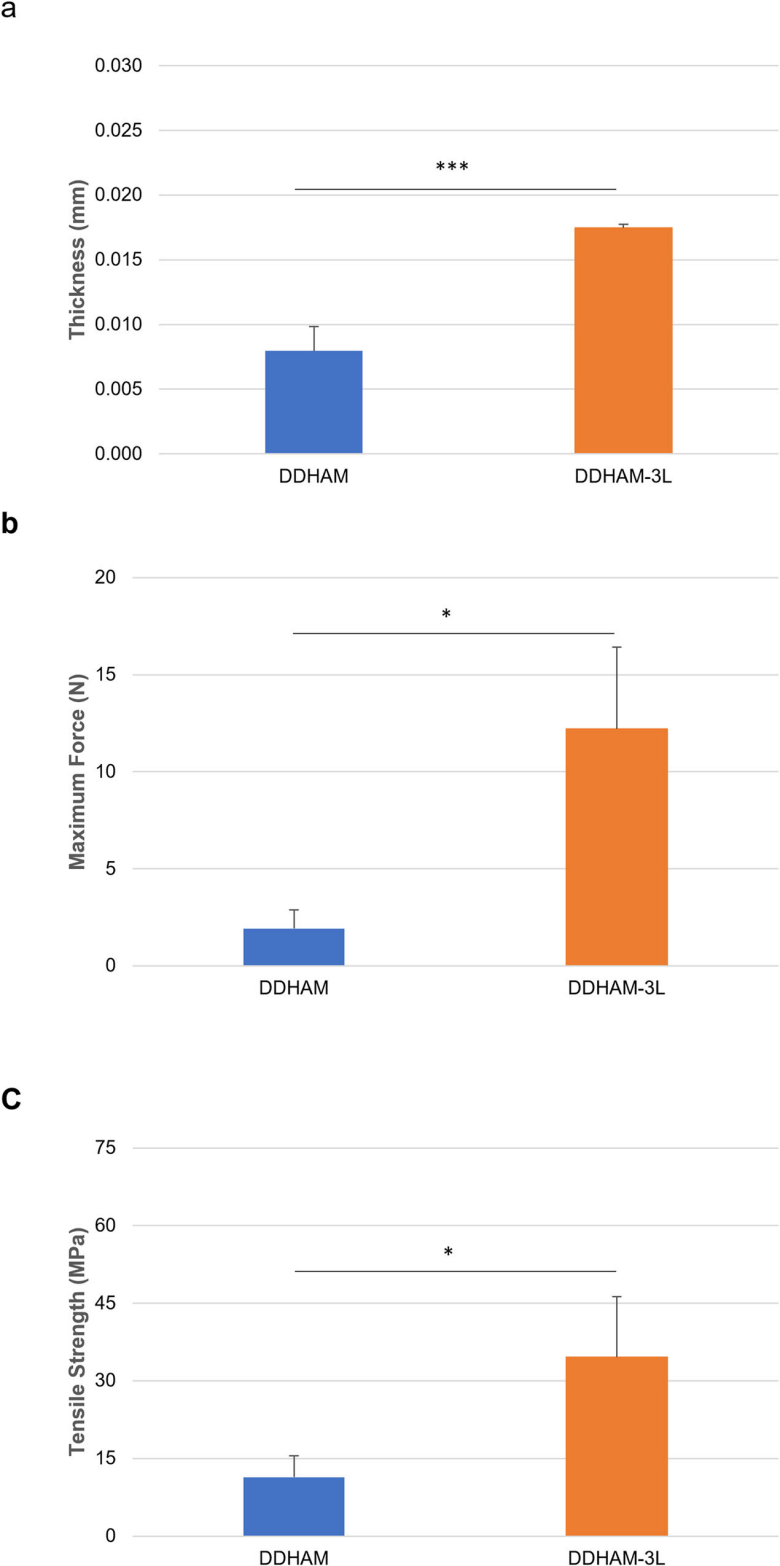
Scaffold characteristics. The average thickness and tensile strength of single-layer (DDHAM) and tri-layer (DDHAM-3L) scaffolds were evaluated. Image (**a**) shows average thickness of DDHAM and DDHAM-3L. Image (**b**) shows maximum force evaluated for DDHAM and DDHAM-3L. Image (**c**) shows the average tensile strength of DDHAM and DDHAM-3L. Data shown are mean ± SD (*n* = 3 biological replicates). *p ≤ 0.05, **p ≤ 0.01, ***p ≤ 0.005. Abbreviations: DDHAM decellularized dehydrated human amniotic membrane, DDHAM-3L tri-layer decellularized dehydrated human amniotic membrane

To better characterize the single-layer and tri-layer scaffolds, the average thickness, width, maximum force, and tensile strength were evaluated. The average thickness was significantly greater for DDHAM-3L (0.017 ± 0.000 mm) than DDHAM (0.008 ± 0.002 mm, *p* < 0.001; Fig. 1a). There was no significant difference in width between the two scaffolds. The maximum force was significantly greater for DDHAM-3L (12.20 ± 4.23 N) than DDHAM (1.92 ± 0.97 N, *p* = 0.015; Fig. 1b). When normalized to width and thickness, the average tensile strength was significantly greater for DDHAM-3L (34.59 ± 11.67 MPa) than DDHAM (11.40 ± 4.14 MPa, *p* = 0.032; Fig. 1c).

**Fig. 2:**
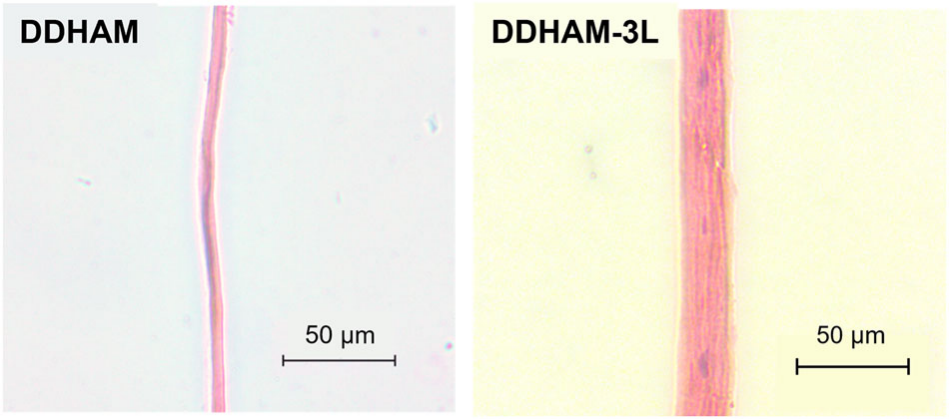
Hematoxylin and Eosin images of DDHAM and DDHAM -3L. The H&E images of DDHAM and DDHAM-3L depict decellularized amniotic membranes devoid of any cells. A decellularized extracellular matrix is depicted within both DDHAM and DDHAM-3L. The darker staining is indicative of the basement membrane in both DDHAM and DDHAM -3L. Scale bar = 50 μm. Abbreviations: DDHAM decellularized dehydrated human amniotic membrane, DDHAM-3L tri-layer decellularized dehydrated human amniotic membrane, H&E Hematoxylin and Eosin, μm micrometer

To evaluate the effect of layering on tissue morphology and to confirm the absence of any residual cells and cell debris, H&E staining was performed on DDHAM and DDHAM-3L (Fig. 2). The surface area depicts preserved morphological features, including a single stromal layer in DDHAM and three integrated layers in DDHAM-3L. The H&E images of both DDHAM and DDHAM-3L demonstrate decellularized AMs devoid of any epithelial cells and cell debris.

### Number of tenocytes on scaffolds

To compare the effects of different scaffolds on tenocytes, tenocytes were seeded to wells containing a scaffold and the number of viable cells was monitored for 1, 2, 4, and 7 days, using alamarBlue assay. Tissue culture treated plastic was used as a control.

**Fig. 3:**
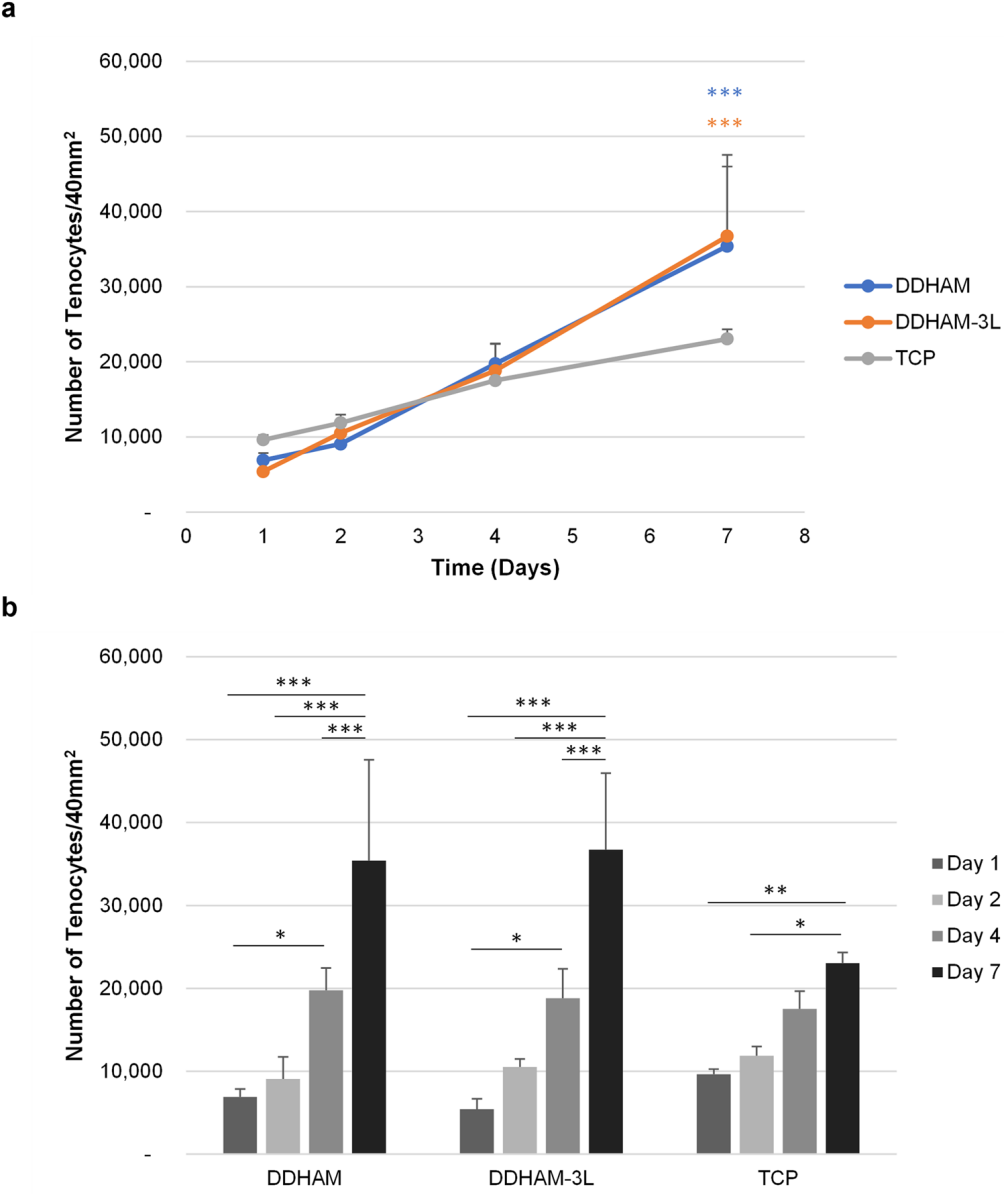
Number of tenocytes on connective tissue matrices over 7 days. Tenocytes were seeded and cultured on different scaffolds. The number of cells was measured by alamarBlue assay on Day 1, Day 2, Day 4, and Day 7. The number of tenocytes on different scaffolds is plotted as both a curve (**a**) and a bar graph (**b**). Data shown are mean ± SD (*n* = 4 biological repeats). A: Asterisk color indicates comparator. Compared with TCP, *p ≤ 0.05, **p ≤ 0.01, ***p ≤ 0.005. Abbreviations: DDHAM decellularized dehydrated human amniotic membrane, DDHAM-3L tri-layer decellularized dehydrated human amniotic membrane, TCP tissue culture treated plastic

Tenocyte growth was observed on all substrates from Day 1 to Day 7 (main effect: time, *p* < 0.001, Fig. 3). There was a significant difference in cell number between scaffolds across time (interaction: scaffold x time, *p* = 0.010). The number of viable cells was similar between DDHAM, DDHAM-3L, and TCP on Day 1, Day 2, and Day 4 (*p* ≥ 0.697). On Day 7, however, the number of cells was significantly greater on DDHAM and DDHAM-3L than on TCP (*p* < 0.001). Thus, the rate of tenocyte proliferation was comparable between DDHAM and DDHAM-3L and was greater than that of cells on TCP (Fig. 3a). This observation suggests that the DDHAM and DDHAM-3L scaffolds promote tenocyte growth. On DDHAM, DDHAM-3L, and TCP, there was a significant increase in the number of cells from Day 1 to Day 7 (*p* < 0.001) and from Day 2 to Day 7 (*p* ≤ 0.024, Fig. 3b). On DDHAM and DDHAM-3L, there was also a significant increase in the number of cells from Day 1 to Day 4 (*p* ≤ 0.025) and from Day 4 to Day 7 (*p* < 0.001).

**Fig. 4:**
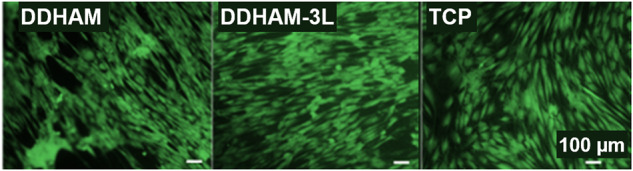
Viable tenocytes on different scaffolds. On Day 7, cells were stained with Calcein AM on the different scaffolds. The images were captured using epi-fluorescent microscope. Representative images of each group are shown. Scale bar = 100 μm. Abbreviations: DDHAM decellularized dehydrated human amniotic membrane, DDHAM-3L tri-layer decellularized dehydrated human amniotic membrane, TCP tissue culture treated plastic, μm micrometer

In addition, when cells were stained with Calcein AM on Day 7, tenocytes showed viable cell growth on DDHAM and DDHAM-3L, which was consistent with the number of cells (Fig. 3). Cells adapted a uni-directionally aligned morphology on the scaffolds but not on TCP (Fig. 4). There was no difference in cell morphology on DDHAM and DDHAM-3L.

### Tenocyte migration

**Fig. 5:**
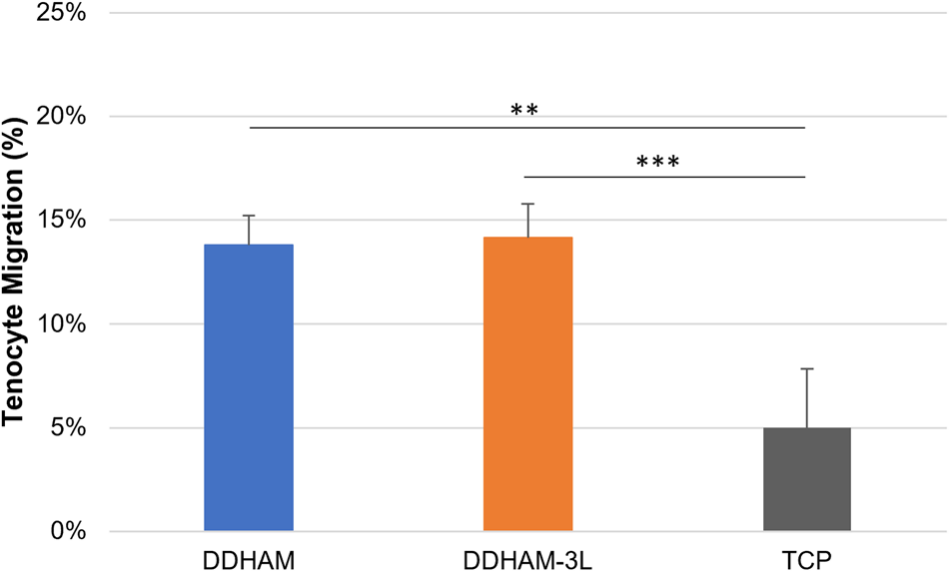
Effects of conditioned media on tenocyte migration. Conditioned medium was collected from cells cultured on scaffolds and TCP after incubation for 24 h. The migration of tenocytes in the presence of conditioned media was monitored using the transwell assay. Tenocyte migration is expressed as the % of migrated cells to the total number of cells. Data shown are mean ± SD (*n* = 3 biological repeats). *p ≤ 0.05, **p ≤ 0.01, ***p ≤ 0.005. Abbreviations: DDHAM decellularized dehydrated human amniotic membrane, DDHAM-3L tri-layer decellularized dehydrated human amniotic membrane, TCP tissue culture treated plastic

To evaluate if tenocytes cultured on different scaffolds release factors to promote cell migration, the CM was collected from cells cultured on different scaffolds. The migration of tenocytes in the presence of such CM was evaluated using a transwell migration assay. Tenocyte migration significantly differed between the different scaffolds (main effect, *p* = 0.003). The CM from cells on DDHAM and DDHAM-3L supported more cell migration compared with CM from the control (*p* = 0.006 and *p* = 0.005, Fig. 5). Tenocytes on scaffolds seem to release factors to promote the migration of tenocytes in the transwell migration assay. There was no significant difference in migration between DDHAM and DDHAM-3L (*p* = 0.996).

### Tenocyte dedifferentiation

**Fig. 6:**
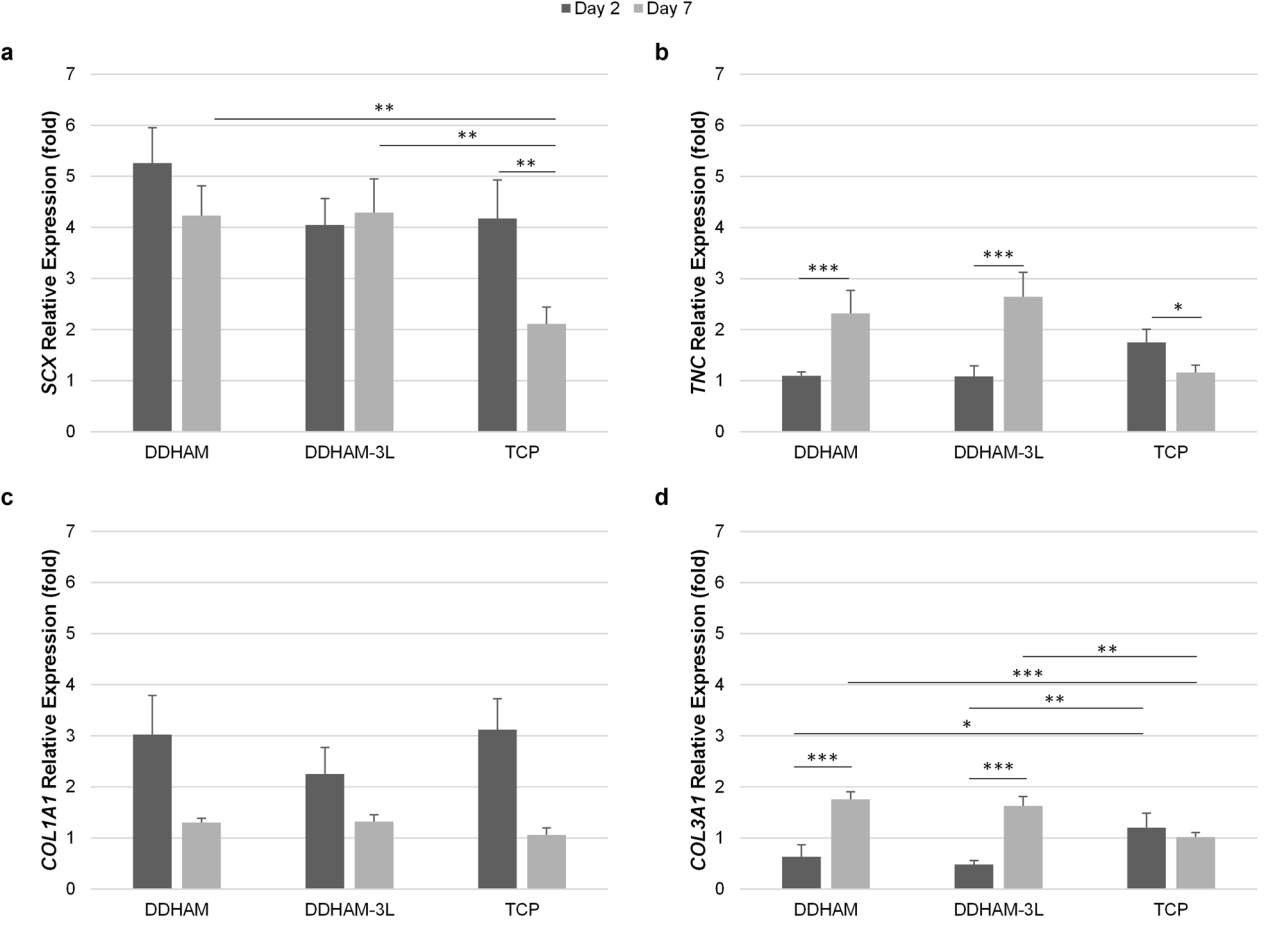
Gene expression of phenotype associated proteins in tenocytes cultured on scaffolds over time. The relative expression of genes associated with tenocytes: *SCX* (**a**), *TNC* (**b**), *COL1A1* (**c**), and *COL3A1* (**d**) in tenocytes cultured on DDHAM, DDHAM-3L, and TCP for 2 and 7 days is shown. The relative expression (fold) was compared to the mRNA level in tenocytes on Day 0 (starting cells). Data shown are mean ± SD (*n* = 3 biological repeats). *p ≤ 0.05, **p ≤ 0.01, ***p ≤ 0.005. Abbreviations: *COL1A1* type I collagen, *COL3A1* type III collagen, DDHAM decellularized dehydrated human amniotic membrane, DDHAM-3L tri-layer decellularized dehydrated human amniotic membrane; *SCX* scleraxis, TCP tissue culture treated plastic, *TNC* tenascin-C

In vitro culturing primary cells often results in dedifferentiation [43]. The presence of an ECM has been shown to reduce the dedifferentiation of primary cells [33, 44]. To evaluate if the presence of scaffolds affects the dedifferentiation of tenocytes, the changes in gene expression of tenocyte phenotype associated proteins were analyzed using qPCR in tenocytes cultured on DDHAM, DDHAM-3L, and TCP (Fig. 6). The mRNA expression of *SCX*, *TNC*, and *COL3A1* varied by scaffold and time (*p* ≤ 0.021). On TCP, the expression of *SCX* and *TNC* significantly declined from Day 2 to Day 7 (*p* ≤ 0.036), and the expression of *COL3A1* remained similar across time (*p* = 0.250). On the other hand, the expression of *SCX* did not significantly change across time when cultured on DDHAM or DDHAM-3L (*p* ≥ 0.061), whereas the expression of *TNC* and *COL3A1* significantly increased from Day 2 to Day 7 (*p* < 0.001). For *COL1A1*, there was a main effect of time (*p* < 0.001), indicating that the expression of *COL1A1* significantly decreased across time.

In addition, on Day 2, mRNA expression of *COL3A1* was significantly higher on TCP than on DDHAM and DDHAM-3L (*p* ≤ 0.008). On Day 7, mRNA expression of *SCX*, *TNC*, and *COL3A1* was significantly greater on DDHAM and DDHAM-3L compared with TCP (*p* ≤ 0.005). Cells on scaffolds seem to maintain gene expression patterns associated with tenocytes better than cells on TCP. There was no difference in gene expression of analyzed genes between DDHAM and DDHAM-3L.

### Inflammatory response at 24 h

**Fig. 7:**
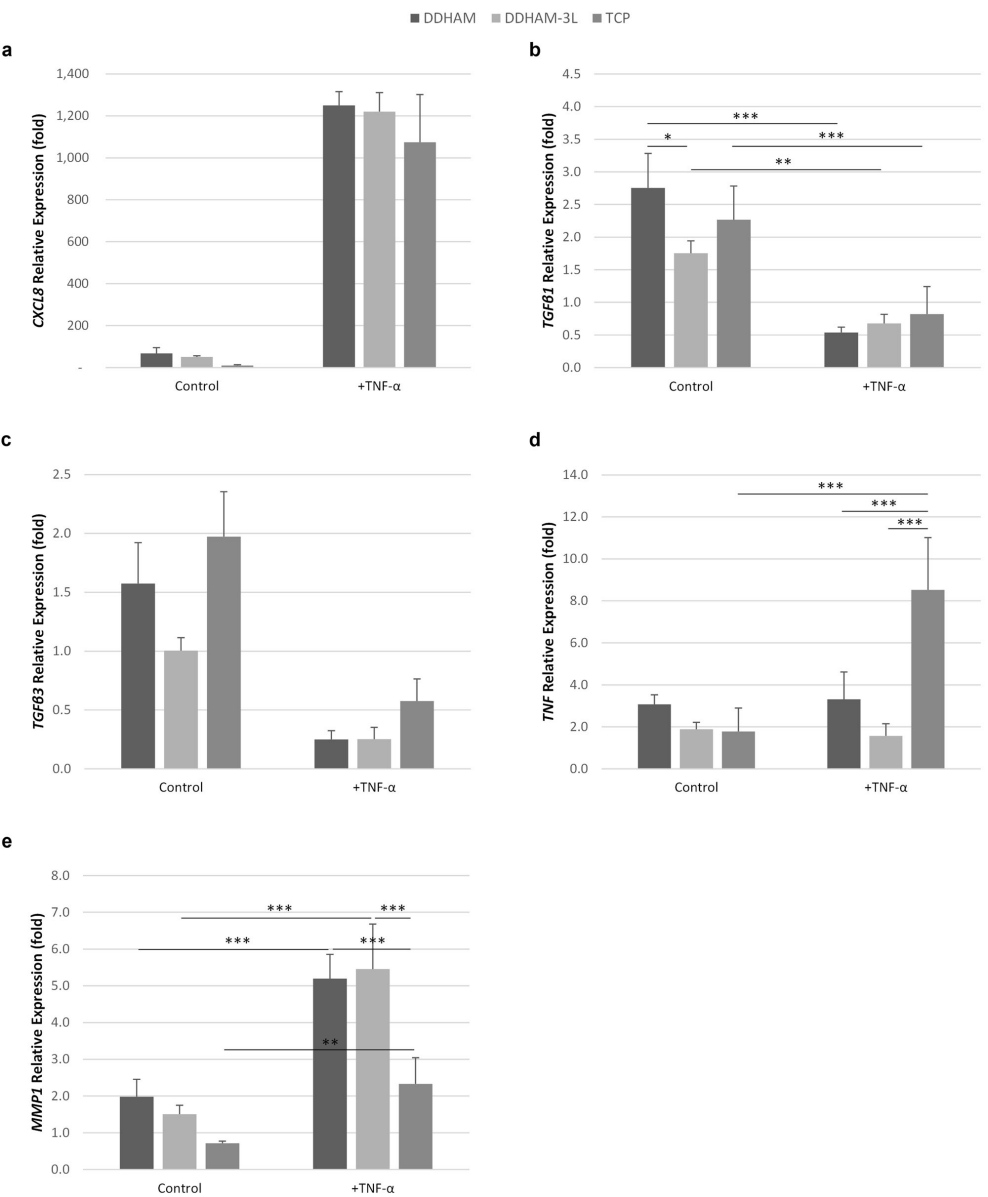
Expression of inflammatory markers in tenocytes cultured on scaffolds with and without stimulation. The relative expression of inflammatory markers in tenocytes cultured on DDHAM, DDHAM-3L, or TCP in stimulated ( + TNF-α) and unstimulated (control) conditions for 24 h is shown. The relative expression (fold) of *CXCL8* (**a**), *TGFβ1* (**b**), *TGFβ3* (**c**), *TNF* (**d**), and *MMP1* (**e**) was compared to the mRNA level in tenocytes on Day 0 (starting cells). Data shown are mean ± SD (*n* = 3 biological repeats). *p < 0.05, **p < 0.01 and ***p < 0.005. Abbreviations: *CXCL8* C-X-C Motif Chemokine Ligand 8, DDHAM decellularized dehydrated human amniotic membrane, DDHAM-3L tri-layer decellularized dehydrated human amniotic membrane, MMP1 matrix metallopeptidase 1, TCP tissue culture treated plastic, *TGFβ1* transforming growth factor beta 1, *TGFβ3* transforming growth factor beta 3

Tenocytes actively participate in tendon repair and regeneration. During the inflammatory stage, tenocytes respond to inflammatory signals and subsequently produce cytokines to modulate inflammation and support tendon regeneration [45]. To determine whether the scaffolds modulate an initial inflammatory response, an inflammatory condition was induced with TNF-α at 24 h. The expression of inflammatory markers (*CXCL8, TNF, TGFβ1, TGFβ3, and MMP1*) in stimulated ( + TNF-α) and unstimulated cells (control), cultured on DDHAM, DDHAM-3L, or TCP were analyzed by qPCR (Fig. 7). Stimulation with TNF-α significantly induced the expression of *CXCL8* (main effect, *p* ≤ 0.001). Conversely, expression of *TGFβ3* significantly declined with TNF-α stimulation (main effect, *p* < 0.001). The expression of *TGFβ1*, *TNF*, and *MMP1* significantly varied by scaffold and stimulation condition (*p* ≤ 0.050). With TNF-α stimulation, the expression of *TGFβ1* significantly declined on all three substrates (*p* ≤ 0.004). Expression of *TNF* significantly increased with TNF-α stimulation on TCP (*p* < 0.001) but not on DDHAM or DDHAM-3L. Stimulation with TNF-α significantly induced the expression of *MMP1* on all three scaffolds (*p* ≤ 0.014).

In the resting state (unstimulated condition), the expression of *TGFβ1* in tenocytes was significantly higher in cells on cultured DDHAM than DDHAM-3L (*p* = 0.018). Under inflammatory conditions, the expression of *MMP1* and *TNF* were significantly higher on TCP than both DDHAM and DDHAM-3L (*p* < 0.001).

There was also a main effect for scaffold, indicating that the mRNA expression of *TGFβ3* was significantly greater on TCP than DDHAM-3L (*p* = 0.001).

## Discussion

Results from the present report demonstrate that the tri-layer design of DDHAM-3L does not alter biological activity of human tenocytes in vitro relative to tenocytes cultured on DDHAM. On Day 1, there was a similar number of viable tenocytes on DDHAM and DDHAM-3L, cell number increased over time on both placental tissue-derived scaffolds, and significantly more tenocyte migration was observed on scaffolds compared with TCP. Moreover, the presence of placental tissue-derived scaffolds reduced tenocyte dedifferentiation and attenuated the inflammatory response of tenocytes in vitro. Collectively, these results suggest that both placental tissue-derived scaffolds may support tendon healing and regeneration in vivo.

At 24 h, cell number was comparable between the two placental tissue-derived scaffolds and TCP. Compared with the standard cell culture surface (TCP), DDHAM and DDHAM-3L supported more cell growth over time. This observation suggests that the placental tissue-derived scaffolds promoted cell proliferation by providing more three-dimensional surface area, by introducing bioactive molecules, or a combination of these two mechanisms [46, 47].

Tenocytes migrate to the site of the injury to begin the healing process. Presumably, an increased number of tenocytes at the site of injury would accelerate the rate of healing [48]. Therefore, the effect of scaffolds on tenocyte migration was evaluated using a transwell assay. The CM of cells on DDHAM and DDHAM-3L supported similar cell migration and significantly more compared with the CM of cells on TCP. This observation suggests that cells cultured on the placental tissue-derived scaffolds may release factors to promote migration. The identity of such factors requires further investigation.

It has been reported previously that the presence of an ECM reduces the dedifferentiation of primary cells [33–35]. To evaluate if the presence of placental tissue-derived scaffolds affects the dedifferentiation of tenocytes, the expression of genes associated with the tenocyte phenotype, cultured on various scaffolds or TCP for 2 and 7 days, were compared using qPCR (Fig. 4). While the expression of *SCX* and *TNC* significantly declined from Day 2 to Day 7 when cultured on TCP, the expression of *SCX*, *TNC*, and *COL3A1* significantly increased or were maintained across time when cultured on both placental tissue-derived scaffolds. This observation suggests that tenocytes cultured on DDHAM and DDHAM-3L showed reduced dedifferentiation in vitro. Contrary to previous findings [35], *COL1A1* declined across time on all scaffolds. This finding suggests that there may have been changes to tenocyte phenotype during this in vitro culture. Despite declines in type I collagen expression, the expression of type III collagen increased over time on both scaffolds. This observation may suggest that the scaffolds support the initiation of the repair response in tenocytes [18].

Although an inflammatory phase is part of the healing process, excessive inflammation can disrupt the phases of repair and impair healing. As a result, the ability to modulate inflammation has been hypothesized as a potential means to improve tendon healing [35, 49]. Recently, placental tissue-derived products have been shown to interact with tenocytes and reduce the inflammatory response [25, 35, 50]. This study sought to evaluate if a tri-layer design altered tenocyte inflammatory gene expression (i.e., *CXCL8*, *TGFβ1*, *TGFβ3*, *TNF*, and *MMP1*). On all substrates, stimulation with TNF-α increased the expression of *CXCL8* and *MMP1* and reduced the expression of *TGFβ1* and *TGFβ3*. Most notably, stimulation with TNF-α significantly increased the expression of *TNF* on TCP but not on the scaffolds. This finding suggests that the presence of placental tissue-derived scaffolds may counteract the pro-inflammatory effect of TNF-α on the expression of *TNF* in tenocytes. In addition, when stimulated with TNF-α, the expression of *MMP1* was higher in cells cultured on scaffolds than on TCP. While interesting, the interpretation of this finding is difficult, as the regulation of *MMP1* is increasingly complex. Although inflammation often induces the expression of *MMP1*, the presence of ECM also stimulates the expression of *MMP1*, even in the absence of inflammation [35, 51].

Previous results have shown that differences in product design influence the cellular activity and inflammatory response of human corneal epithelial cells [40]. For this reason, we hypothesized that differences in product design would influence the biological activity of the scaffolds. Unlike the previous study, however, the present report selected two products that share identical processing and decellularization protocols. Therefore, the primary difference between the two placental tissue-derived products was the tri-layer design. It is important to note, however, DDHAM-3L is uniquely designed with an all-sided stromal interface, regardless of its orientation, whereas DDHAM possesses both the epithelial and stromal layers, permitting use in either orientation. Although the single layer DDHAM possesses both an epithelial and a stromal side, it’s designed as a non-side specific membrane. Therefore, the sidedness was not evaluated in this study. To better characterize the single-layer and tri-layer scaffolds, the present report conducted tensile testing and performed H&E staining. The average thickness of DDHAM-3L was found to be twice that of DDHAM. For identically sized biomaterials, DDHAM-3L was approximately six times stronger than DDHAM. Thus, when the maximum force was normalized to thickness, the tensile strength of DDHAM-3L was three-fold that of DDHAM. Furthermore, H&E staining confirmed the absence of residual cells and cell debris and demonstrated preserved morphological features, including a single stromal layer in DDHAM and three integrated layers in DDHAM-3L. Collectively, these results demonstrate that the tri-layer scaffold possesses increased handling properties with preservation of tissue morphology. Moreover, the tensile strength of both DDHAM (11.40 ± 4.15 MPa) and DDHAM-3L (34.58 ± 11.67 MPa) is notably higher than the reported tensile strength of native human amniotic membrane (≤6.80 ± 0.22 MPa) [52, 53].

However, this study is not without limitation. The tensile strength values are approximate and can vary depending on the specific study, testing methodology, and sample preparation. Additionally, the tensile strength values of amniotic membranes can be influenced by factors such as donor variability, tissue thickness, and processing conditions. Moreover, in this series of experiments, human tenocytes derived from healthy donors were used to examine the role of scaffolds in tendon repair and regeneration. As previously acknowledged [35], cells respond differently when derived from healthy or diseased tissue [54–56]. Therefore, the effects of scaffolds on cells isolated from diseased tissues must also be investigated. In addition, this in vitro study only used one cell type. In vivo, additional cells interact with the tenocytes (e.g., macrophages), which may influence cell-scaffold interactions. The authors also identified and examined several inflammatory markers, known to be present during the inflammatory phase of tendon healing [9, 21–24]. However, this list is not exhaustive and other pro-inflammatory mediators (e.g., *IL6*, *IL1*) should be considered for a more complete understanding of how scaffolds modulate the inflammatory response. Although gene expression has been used to evaluate tenocyte dedifferentiation in other published reports [35, 50, 57], confirming gene expression with protein levels would better elucidate the biological significance of these findings. Additionally, the genes used to evaluate differentiation are non-specific and are expressed by many other musculoskeletal tissues. Lastly, and perhaps most importantly, the results of this simplified in vitro study may not equate to the results associated with the clinical application of these scaffolds. Comparative studies examining the outcomes associated with the clinical application of these scaffolds in tendon repair and regeneration are warranted.

## Conclusions

This in vitro study demonstrated that a tri-layer design did not alter the biological activity of human tenocytes in vitro. DDHAM and DDHAM-3L similarly support tenocyte function. The presence of DDHAM and DDHAM-3L was associated with significantly greater cell growth and cell migration, while also attenuating tenocyte dedifferentiation and the inflammatory response. However, a comparative investigation is needed to more fully evaluate whether these differences in product design influence clinical outcomes.

## Supplementary information

Supplemental Material

## Data Availability

Research data is included as electronic Supplementary Information.
