# Supplementary material for: A tri-layer decellularized, dehydrated human amniotic membrane scaffold supports the cellular functions of human tenocytes in vitro
Source: J Mater Sci Mater Med. 2023 Jul 24;34(7):37. doi: 10.1007/s10856-023-06740-4 (PMC10366303; doi:10.1007/s10856-023-06740-4)
Supplement: Supplementary file 1 — Supplemental Material [file 10856_2023_6740_MOESM1_ESM.docx]

**Supplemental Material**

**Data Set 1** Tensile Strength

| **Scaffold** | **Reading** | **Thickness(mm)** | **Width (mm)** | **Maximum Force (N)** | **Tensile Strength (MPa)** |
| --- | --- | --- | --- | --- | --- |
| DDHAM | 1 | 0.0082 | 21.0 | 2.55 | 14.94 |
| DDHAM | 2 | 0.0060 | 19.5 | 0.8 | 6.84 |
| DDHAM | 3 | 0.0097 | 20.0 | 2.4 | 12.41 |
| DDHAM-3L | 1 | 0.0178 | 20.0 | 15.4 | 43.38 |
| DDHAM-3L | 2 | 0.0173 | 20.4 | 13.8 | 39.03 |
| DDHAM-3L | 3 | 0.0173 | 20.0 | 7.4 | 21.35 |

**Data Set 2** Cell Number

| **Scaffold** | **Reading** | **Time (Day)** | **Cell Number** |
| --- | --- | --- | --- |
| DDHAM | 1 | 1 | 7,828 |
| DDHAM | 2 | 1 | 6,112 |
| DDHAM | 3 | 1 | 6,088 |
| DDHAM | 4 | 1 | 7,646 |
| DDHAM-3L | 1 | 1 | 5,901 |
| DDHAM-3L | 2 | 1 | 5,297 |
| DDHAM-3L | 3 | 1 | 6,721 |
| DDHAM-3L | 4 | 1 | 3,734 |
| TCP | 1 | 1 | 10,080 |
| TCP | 2 | 1 | 10,190 |
| TCP | 3 | 1 | 8,867 |
| TCP | 4 | 1 | 9,411 |
| DDHAM | 1 | 2 | 11,731 |
| DDHAM | 2 | 2 | 5,474 |
| DDHAM | 3 | 2 | 8,893 |
| DDHAM | 4 | 2 | 10,154 |
| DDHAM-3L | 1 | 2 | 9,986 |
| DDHAM-3L | 2 | 2 | 9,924 |
| DDHAM-3L | 3 | 2 | 10,250 |
| DDHAM-3L | 4 | 2 | 11,933 |
| TCP | 1 | 2 | 11,692 |
| TCP | 2 | 2 | 10,849 |
| TCP | 3 | 2 | 11,554 |
| TCP | 4 | 2 | 13,433 |
| DDHAM | 1 | 4 | 23,186 |
| DDHAM | 2 | 4 | 20,592 |
| DDHAM | 3 | 4 | 17,403 |
| DDHAM | 4 | 4 | 17,835 |
| DDHAM-3L | 1 | 4 | 24,102 |
| DDHAM-3L | 2 | 4 | 17,408 |
| DDHAM-3L | 3 | 4 | 16,444 |
| DDHAM-3L | 4 | 4 | 17,317 |
| TCP | 1 | 4 | 20,220 |
| TCP | 2 | 4 | 17,862 |
| TCP | 3 | 4 | 16,977 |
| TCP | 4 | 4 | 15,038 |
| DDHAM | 1 | 7 | 30,795 |
| DDHAM | 2 | 7 | 22,486 |
| DDHAM | 3 | 7 | 22,217 |
| DDHAM | 4 | 7 | 24,859 |
| DDHAM | 5 | 7 | 54,749 |
| DDHAM | 6 | 7 | 39,176 |
| DDHAM | 7 | 7 | 42,134 |
| DDHAM | 8 | 7 | 46,747 |
| DDHAM-3L | 1 | 7 | 30,119 |
| DDHAM-3L | 2 | 7 | 25,765 |
| DDHAM-3L | 3 | 7 | 28,455 |
| DDHAM-3L | 4 | 7 | 31,231 |
| DDHAM-3L | 5 | 7 | 46,872 |
| DDHAM-3L | 6 | 7 | 49,504 |
| DDHAM-3L | 7 | 7 | 45,078 |
| DDHAM-3L | 8 | 7 | 36,803 |
| TCP | 1 | 7 | 24,846 |
| TCP | 2 | 7 | 21,218 |
| TCP | 3 | 7 | 21,930 |
| TCP | 4 | 7 | 24,163 |
| TCP | 5 | 7 | 23,157 |
| TCP | 6 | 7 | 23,597 |
| TCP | 7 | 7 | 21,752 |
| TCP | 8 | 7 | 23,759 |

**Data Set 3** Cell Migration

| **Scaffold** | **Reading** | **Migration** |
| --- | --- | --- |
| DDHAM | 1 | 0.12 |
| DDHAM | 2 | 0.14 |
| DDHAM | 3 | 0.15 |
| DDHAM-3L | 1 | 0.15 |
| DDHAM-3L | 2 | 0.15 |
| DDHAM-3L | 3 | 0.12 |
| TCP | 1 | 0.07 |
| TCP | 2 | 0.06 |
| TCP | 3 | 0.02 |

**Data Set 4** Tenocyte Dedifferentiation

| **Scaffold** | **Reading** | **Time (Day)** | **Gene** | **mRNA Expression** |
| --- | --- | --- | --- | --- |
| DDHAM | 1 | 2 | *SCX* | 6.04 |
| DDHAM | 2 | 2 | *SCX* | 4.73 |
| DDHAM | 3 | 2 | *SCX* | 4.99 |
| DDHAM-3L | 1 | 2 | *SCX* | 4.25 |
| DDHAM-3L | 2 | 2 | *SCX* | 3.46 |
| DDHAM-3L | 3 | 2 | *SCX* | 4.43 |
| TCP | 1 | 2 | *SCX* | 3.60 |
| TCP | 2 | 2 | *SCX* | 3.90 |
| TCP | 3 | 2 | *SCX* | 5.03 |
| DDHAM | 1 | 7 | *SCX* | 4.87 |
| DDHAM | 2 | 7 | *SCX* | 3.71 |
| DDHAM | 3 | 7 | *SCX* | 4.11 |
| DDHAM-3L | 1 | 7 | *SCX* | 5.05 |
| DDHAM-3L | 2 | 7 | *SCX* | 3.89 |
| DDHAM-3L | 3 | 7 | *SCX* | 3.93 |
| TCP | 1 | 7 | *SCX* | 1.91 |
| TCP | 2 | 7 | *SCX* | 2.49 |
| TCP | 3 | 7 | *SCX* | 1.93 |
| DDHAM | 1 | 2 | *TNC* | 1.17 |
| DDHAM | 2 | 2 | *TNC* | 1.09 |
| DDHAM | 3 | 2 | *TNC* | 1.02 |
| DDHAM-3L | 1 | 2 | *TNC* | 1.29 |
| DDHAM-3L | 2 | 2 | *TNC* | 0.89 |
| DDHAM-3L | 3 | 2 | *TNC* | 1.07 |
| TCP | 1 | 2 | *TNC* | 1.54 |
| TCP | 2 | 2 | *TNC* | 1.68 |
| TCP | 3 | 2 | *TNC* | 2.04 |
| DDHAM | 1 | 7 | *TNC* | 2.44 |
| DDHAM | 2 | 7 | *TNC* | 1.82 |
| DDHAM | 3 | 7 | *TNC* | 2.70 |
| DDHAM-3L | 1 | 7 | *TNC* | 2.41 |
| DDHAM-3L | 2 | 7 | *TNC* | 2.33 |
| DDHAM-3L | 3 | 7 | *TNC* | 3.19 |
| TCP | 1 | 7 | *TNC* | 1.09 |
| TCP | 2 | 7 | *TNC* | 1.07 |
| TCP | 3 | 7 | *TNC* | 1.33 |
| DDHAM | 1 | 2 | *COL1A1* | 3.67 |
| DDHAM | 2 | 2 | *COL1A1* | 3.23 |
| DDHAM | 3 | 2 | *COL1A1* | 2.17 |
| DDHAM-3L | 1 | 2 | *COL1A1* | 2.27 |
| DDHAM-3L | 2 | 2 | *COL1A1* | 1.72 |
| DDHAM-3L | 3 | 2 | *COL1A1* | 2.76 |
| TCP | 1 | 2 | *COL1A1* | 2.55 |
| TCP | 2 | 2 | *COL1A1* | 3.05 |
| TCP | 3 | 2 | *COL1A1* | 3.76 |
| DDHAM | 1 | 7 | *COL1A1* | 1.25 |
| DDHAM | 2 | 7 | *COL1A1* | 1.25 |
| DDHAM | 3 | 7 | *COL1A1* | 1.40 |
| DDHAM-3L | 1 | 7 | *COL1A1* | 1.47 |
| DDHAM-3L | 2 | 7 | *COL1A1* | 1.25 |
| DDHAM-3L | 3 | 7 | *COL1A1* | 1.24 |
| TCP | 1 | 7 | *COL1A1* | 0.93 |
| TCP | 2 | 7 | *COL1A1* | 1.05 |
| TCP | 3 | 7 | *COL1A1* | 1.20 |
| DDHAM | 1 | 2 | *COL3A1* | 0.84 |
| DDHAM | 2 | 2 | *COL3A1* | 0.67 |
| DDHAM | 3 | 2 | *COL3A1* | 0.38 |
| DDHAM-3L | 1 | 2 | *COL3A1* | 0.54 |
| DDHAM-3L | 2 | 2 | *COL3A1* | 0.39 |
| DDHAM-3L | 3 | 2 | *COL3A1* | 0.51 |
| TCP | 1 | 2 | *COL3A1* | 0.93 |
| TCP | 2 | 2 | *COL3A1* | 1.17 |
| TCP | 3 | 2 | *COL3A1* | 1.50 |
| DDHAM | 1 | 7 | *COL3A1* | 1.73 |
| DDHAM | 2 | 7 | *COL3A1* | 1.62 |
| DDHAM | 3 | 7 | *COL3A1* | 1.92 |
| DDHAM-3L | 1 | 7 | *COL3A1* | 1.70 |
| DDHAM-3L | 2 | 7 | *COL3A1* | 1.42 |
| DDHAM-3L | 3 | 7 | *COL3A1* | 1.76 |
| TCP | 1 | 7 | *COL3A1* | 0.91 |
| TCP | 2 | 7 | *COL3A1* | 1.05 |
| TCP | 3 | 7 | *COL3A1* | 1.09 |

**Data Set 5** Inflammatory Response

| **Scaffold** | **Reading** | **Stimulation Condition** | **Gene** | **mRNA Expression** |
| --- | --- | --- | --- | --- |
| DDHAM | 1 | Control | *CXCL8* | 97.33 |
| DDHAM | 2 | Control | *CXCL8* | 61.36 |
| DDHAM | 3 | Control | *CXCL8* | 42.86 |
| DDHAM-3L | 1 | Control | *CXCL8* | 45.41 |
| DDHAM-3L | 2 | Control | *CXCL8* | 50.33 |
| DDHAM-3L | 3 | Control | *CXCL8* | 56.17 |
| TCP | 1 | Control | *CXCL8* | 5.88 |
| TCP | 2 | Control | *CXCL8* | 11.22 |
| TCP | 3 | Control | *CXCL8* | 13.23 |
| DDHAM | 1 | +TNF-α | *CXCL8* | 1196.04 |
| DDHAM | 2 | +TNF-α | *CXCL8* | 1323.12 |
| DDHAM | 3 | +TNF-α | *CXCL8* | 1230.16 |
| DDHAM-3L | 1 | +TNF-α | *CXCL8* | 1203.15 |
| DDHAM-3L | 2 | +TNF-α | *CXCL8* | 1318.20 |
| DDHAM-3L | 3 | +TNF-α | *CXCL8* | 1140.53 |
| TCP | 1 | +TNF-α | *CXCL8* | 1006.87 |
| TCP | 2 | +TNF-α | *CXCL8* | 1327.72 |
| TCP | 3 | +TNF-α | *CXCL8* | 887.87 |
| DDHAM | 1 | Control | *TGFβ1* | 2.75 |
| DDHAM | 2 | Control | *TGFβ1* | 3.28 |
| DDHAM | 3 | Control | *TGFβ1* | 2.23 |
| DDHAM-3L | 1 | Control | *TGFβ1* | 1.56 |
| DDHAM-3L | 2 | Control | *TGFβ1* | 1.77 |
| DDHAM-3L | 3 | Control | *TGFβ1* | 1.94 |
| TCP | 1 | Control | *TGFβ1* | 1.78 |
| TCP | 2 | Control | *TGFβ1* | 2.21 |
| TCP | 3 | Control | *TGFβ1* | 2.81 |
| DDHAM | 1 | +TNF-α | *TGFβ1* | 0.59 |
| DDHAM | 2 | +TNF-α | *TGFβ1* | 0.58 |
| DDHAM | 3 | +TNF-α | *TGFβ1* | 0.44 |
| DDHAM-3L | 1 | +TNF-α | *TGFβ1* | 0.76 |
| DDHAM-3L | 2 | +TNF-α | *TGFβ1* | 0.76 |
| DDHAM-3L | 3 | +TNF-α | *TGFβ1* | 0.52 |
| TCP | 1 | +TNF-α | *TGFβ1* | 0.63 |
| TCP | 2 | +TNF-α | *TGFβ1* | 1.30 |
| TCP | 3 | +TNF-α | *TGFβ1* | 0.53 |
| DDHAM | 1 | Control | *TGFβ3* | 1.97 |
| DDHAM | 2 | Control | *TGFβ3* | 1.41 |
| DDHAM | 3 | Control | *TGFβ3* | 1.34 |
| DDHAM-3L | 1 | Control | *TGFβ3* | 0.93 |
| DDHAM-3L | 2 | Control | *TGFβ3* | 0.95 |
| DDHAM-3L | 3 | Control | *TGFβ3* | 1.13 |
| TCP | 1 | Control | *TGFβ3* | 1.78 |
| TCP | 2 | Control | *TGFβ3* | 1.73 |
| TCP | 3 | Control | *TGFβ3* | 2.41 |
| DDHAM | 1 | +TNF-α | *TGFβ3* | 0.31 |
| DDHAM | 2 | +TNF-α | *TGFβ3* | 0.27 |
| DDHAM | 3 | +TNF-α | *TGFβ3* | 0.17 |
| DDHAM-3L | 1 | +TNF-α | *TGFβ3* | 0.34 |
| DDHAM-3L | 2 | +TNF-α | *TGFβ3* | 0.27 |
| DDHAM-3L | 3 | +TNF-α | *TGFβ3* | 0.14 |
| TCP | 1 | +TNF-α | *TGFβ3* | 0.60 |
| TCP | 2 | +TNF-α | *TGFβ3* | 0.75 |
| TCP | 3 | +TNF-α | *TGFβ3* | 0.38 |
| DDHAM | 1 | Control | *TNF* | 3.11 |
| DDHAM | 2 | Control | *TNF* | 3.51 |
| DDHAM | 3 | Control | *TNF* | 2.61 |
| DDHAM-3L | 1 | Control | *TNF* | 1.54 |
| DDHAM-3L | 2 | Control | *TNF* | 1.92 |
| DDHAM-3L | 3 | Control | *TNF* | 2.19 |
| TCP | 1 | Control | *TNF* | 1.42 |
| TCP | 2 | Control | *TNF* | 3.03 |
| TCP | 3 | Control | *TNF* | 0.90 |
| DDHAM | 1 | +TNF-α | *TNF* | 2.24 |
| DDHAM | 2 | +TNF-α | *TNF* | 4.77 |
| DDHAM | 3 | +TNF-α | *TNF* | 2.92 |
| DDHAM-3L | 1 | +TNF-α | *TNF* | 1.49 |
| DDHAM-3L | 2 | +TNF-α | *TNF* | 1.03 |
| DDHAM-3L | 3 | +TNF-α | *TNF* | 2.19 |
| TCP | 1 | +TNF-α | *TNF* | 6.34 |
| TCP | 2 | +TNF-α | *TNF* | 11.24 |
| TCP | 3 | +TNF-α | *TNF* | 7.99 |
| DDHAM | 1 | Control | *MMP1* | 1.83 |
| DDHAM | 2 | Control | *MMP1* | 2.51 |
| DDHAM | 3 | Control | *MMP1* | 1.60 |
| DDHAM-3L | 1 | Control | *MMP1* | 1.43 |
| DDHAM-3L | 2 | Control | *MMP1* | 1.30 |
| DDHAM-3L | 3 | Control | *MMP1* | 1.78 |
| TCP | 1 | Control | *MMP1* | 0.65 |
| TCP | 2 | Control | *MMP1* | 0.71 |
| TCP | 3 | Control | *MMP1* | 0.77 |
| DDHAM | 1 | +TNF-α | *MMP1* | 4.80 |
| DDHAM | 2 | +TNF-α | *MMP1* | 5.96 |
| DDHAM | 3 | +TNF-α | *MMP1* | 4.84 |
| DDHAM-3L | 1 | +TNF-α | *MMP1* | 5.60 |
| DDHAM-3L | 2 | +TNF-α | *MMP1* | 6.60 |
| DDHAM-3L | 3 | +TNF-α | *MMP1* | 4.16 |
| TCP | 1 | +TNF-α | *MMP1* | 1.93 |
| TCP | 2 | +TNF-α | *MMP1* | 3.15 |
| TCP | 3 | +TNF-α | *MMP1* | 1.91 |
